# Nd-YAG Laser Treatment for Tracheobronchial Obstruction

**DOI:** 10.1155/DTE.3.107

**Published:** 1996

**Authors:** Reury-Perng Perng, Yu-Chin Lee, Kuo-Hwa Chiang

**Affiliations:** Chest Department Veterans General Hospital-Taipei 201 Shih-Pai Road, Section 2 Taipei Taiwan 11217 China

## Abstract

The Nd-YAG laser has good tissue penetration and coagulation effects thus has become an important weapon for photoresection of tracheobronchial obstructive lesions since 1980.

Treatment of benign lesions including benign tumors and scar tissues using the Nd-YAG laser has good results. In the treatment of malignant tumors however, it has a lower effectivity rate when compared to benign lesions. From July 1984 to September 1995, a total of 65 patients were treated with Nd-YAG laser for tracheobronchial obstruction. There were 32 (49%) malignant tumors and 33 (51%) benign lesions. 116 resections were performed in 48 patients using the non-contact Nd-YAG laser (MBB, Medilas 2) before 1992. Thereafter, another 41 resections were performed in 17 cases using contact Nd-YAG laser (SLT, CL-X). The overall effectivity rate was 60%. The effectivity rate for benign lesions was 81.3% and 39.4% for malignant tumor. The effectivity rate between non-contact and contact Nd-YAG laser was not significantly different.


*Diagnostic and Therapeutic Endoscopy*, 1996, Vol. 3, pp. 107–110
Reprints available directly from the publisher
Photocopying permitted by license only

© 1996 OPA (Overseas Publishers Association)
Amsterdam B.V. Published in The Netherlands
by Harwood Academic Publishers
Printed in Malaysia

### Nd-YAG Laser Treatment for Tracheobronchial Obstruction

REURY-PERNG PERNG*, YU-CHIN LEE, and KUO-HWA CHIANG

*Chest Department, Veterans General Hospital-Taipei, 201 Shih-Pai Road, Section 2, Taipei, Taiwan 11217, Republic of China*

*(Received 11 June 1996; In final form 26 June 1996)*

**The Nd-YAG laser has good tissue penetration and coagulation effects thus has become an important weapon for photoresection of tracheobronchial obstructive lesions since 1980.**

**Treatment of benign lesions including benign tumors and scar tissues using the Nd-YAG laser has good results. In the treatment of malignant tumors however, it has a lower effectivity rate when compared to benign lesions. From July 1984 to September 1995, a total of 65 patients were treated with Nd-YAG laser for tracheobronchial obstruction. There were 32 (49%) malignant tumors and 33 (51%) benign lesions. 116 resections were performed in 48 patients using the non-contact Nd-YAG laser (MBB, Medilas 2) before 1992. Thereafter, another 41 resections were performed in 17 cases using contact Nd-YAG laser (SLT, CL-X). The overall effectivity rate was 60%. The effectivity rate for benign lesions was 81.3% and 39.4% for malignant tumor. The effectivity rate between non-contact and contact Nd-YAG laser was not significantly different.**

*Keywords:* Nd-YAG laser, tracheobronchial obstruction, treatment

### INTRODUCTION

The causes of tracheobronchial obstruction are divided into two main groups as to the nature of the occluding lesion. One is malignant neogrowths, primary as well as metastatic tumors, or it may be benign, like scar tissues, benign tumors, foreign body and others.

Tracheobronchial obstruction can be treated either by surgery, radiotherapy, cryosurgery, electrocoagulation, laser photoresection, photodynamic therapy or by placement of tracheal stents for relief of obstruction. Laser treatment for airway obstruction secondary to malignant tumor is mainly palliative. $CO_2$, Argon and Nd-YAG laser are mediums for laser photoresection. The most effective medium is Nd-YAG due to its greater power density and deeper degree of tissue penetration. Laser treatment may sometimes be used for the curative treatment of small superficial lesions of the tracheobronchial tree and also for low-grade malignant tumors. Utilization of laser photoresection for the palliative treatment of malignant tumors have several advantages compared to other modalities of treatment. Firstly, there is no systemic toxicity secondary to cumulation of doses as in radiotherapy.

*Corresponding author. Tel: 886-2-875-7562. Fax: 886-2-875-2380.

Furthermore, the procedure is well-tolerated by the patient, attains adequate hemostasis and causes little damage to surrounding tissues. $CO_2$, Argon and Nd-YAG laser photoresection can also be used for the treatment of airway obstruction due to benign lesions. As in cases of malignancy, the Nd-YAG laser is the most effective. Generally speaking, there are better results and lesser complications in cases of benign lesion than in malignant tumor [1]. Scar tissues may cause diaphragmatic or bottle-neck types of airway obstruction. Treatment outcomes are better for the diaphragmatic type than for the bottle-neck types [2].

Using laser photoresection for treatment of airway obstruction poses some limitations. Lesions should be intraluminal and should be within the accessible range of the bronchoscope. Conditions such as upper lobe bronchus obstruction and obstruction length more than 4 cm. makes the treatment technically difficult and thus, have poor results. The complications of laser photoresection are: massive bleeding, perforation of bronchial wall, bronchial obstruction, smoke inhalation and others such as cardiac arrest and arrhythmias.

A rigid or a flexible bronchoscope is generally employed for laser photoresection. Advantages and disadvantages for each type of bronchoscope is shown in Table I. In general, rigid bronchoscope is used for obstructions in the central airways associated with ventilatory distress and the flexible bronchoscope is used for distal small airways obstruction. Nd-YAG laser for photoresection are of two types, contact and non-contact. Contact Nd-YAG laser probes are made of synthetic sapphire crystal which was available since the early 1980s [3]. Contact Nd-YAG laser offers several advantages including: greater precision in aiming, lower power requirement, greater control of depth penetration and more protection of the laser fiber from soiling and deterioration. From 1984, the Nd-YAG laser became a treatment option for tracheobronchial obstruction at the Veteran General Hospital-Taipei. We collected all cases of laser photoresection performed at our institution over the past 10 years and data were analyzed retrospectively.

The purposes of this study are: (1) to determine the indications and utilities of Nd-YAG laser treatment, (2) to evaluate the treatment results of different disease groups, (3) to compare results between contact and non-contact Nd-YAG laser treatments and (4) to evaluate the possible complications of Nd-YAG laser treatment.

### MATERIALS AND METHODS

From July 1984 to September 1995, there were 65 patients who received Nd-YAG laser treatment for tracheobronchial obstruction. A total of 157 photoresections were performed. One hundred sixteen photoresections for 48 patients were performed using the non-contact Nd-YAG laser and 41 photoresections for 17 patients were performed using the contact Nd-YAG laser. During treatment of these patients, we employed the use of a flexible fiberoptic bronchoscope and performed the treatment under local anaesthesia. Prior to 1992, we used non-contact type Nd-YAG laser (MBB, Medilas 2), afterwhich, we changed to the use of contact type Nd-YAG laser (SLT, CL-X).

The diagnoses of the study group are shown in Table II. Benign lesions comprised 49%, mostly scar tissues. Malignant tumor accounted for 51%, which are most commonly situated in the bronchus.

TABLE I Comparison between rigid and flexible bronchoscope in laser photoresection

| | Rigid | Flexible |
|---|---|---|
| General anaesthesia risk | Yes | No |
| Ease of manipulation | No | Yes |
| Bleeding control | Good | Poor |
| Fire risk | No | Yes |
| Resectability | Good | Poor |
| Airway control | Good | Poor |

TABLE II Distribution of cases

| Diagnosis | No. | % |
|---|---|---|
| Benign | 32 | 49 |
| Scar tissue | 23 | |
| Tumor | 7 | |
| Others | 2 | |
| Malignant | 33 | 51 |
| Trachea | 7 | |
| Bronchus | 23 | |
| Others | 3 | |

### RESULTS

The effectivity rates of laser treatment for different diagnostic groups are shown in Table III. The overall effectivity rate for benign lesions is 81.3%, better than for malignant tumor with only 39.4% ($p < 0.005$). About half of benign cases were cured by laser treament alone. As a whole, we had an effectivity rate of 60%.

The effectivity rate of laser treatment as to types of lasers are shown in Table IV. The effectivity rate for non-contact laser is 62.5% and 52.9% for contact laser, showing no significant difference ($p > 0.4$). We encountered only 3 cases of major complications which included one case of massive bleeding, one case of cardiac arrhythmia and one case of pneumonia. No procedure-related mortality occurred.

### DISCUSSION

The effectivity rate of Nd-YAG laser treatment for tracheobronchial obstruction by malignant tumor is around 42.7 to 84.4% [4,5]. It is mainly determined by the following factors: criteria of patient selection, experience of the bronchoscopist, type of bronchoscope used, and type of anaesthesia. A rather aggressive attitude with employment of rigid bronchoscope with good coordination between the bronchoscopist and anaesthesiologist would probably have yielded a higher effectivity rate with lesser complications. Usage of the flexible bronchoscope and local anaesthesia, as in our patient group had some shortcomings. Patient cooperation was extremely vital and results varied subjectively. The short anaesthesia duration posed another problem. In addition, evacuation of necrotic tissues was often incomplete, bleeding control inadequate and control of ventilation was difficult and far from ideal. These could have accounted for a rather low (39.4%) effectivity rate of Nd-YAG laser treatment of airway obstruction, due to malignant tumor. We had one case of intraluminal carcinoid of low grade malignancy with complete cure by laser treatment alone and had no evidence of recurrence up to 5 years follow-up. The effectivity rate of Nd-YAG laser treatment for tracheobronchial obstruction by benign lesions is usually better than that caused by malignant tumor. In our series, the effectivity rate was 81.3% for benign lesions compared to 39.4% for malignant tumor. Although there were some advantages of contact Nd-YAG laser than the non-contact type, the effectivity rates for both types of Nd-YAG lasers were not significantly different ($p > 0.4$). The small sample size in this series could have made this finding weak. No comparison of side effects was made in this study. If there was, there would be expectedly, lesser complication with the contact type Nd-YAG laser. We had 3 cases with major complications and no procedure-related mortality. Reported incidences of morbidity in the literature ranged from 1.8 to 2.3%, and the mortality rate was 0.4 to 1.2% [4–11].

TABLE III Effectivity rate of ND-YAG laser treatment according to diagnosis type

| Diagnosis | Patient no. | C.E. (%) | P.E. (%) | Overall (%) |
|---|---|---|---|---|
| Benign | 32 | 40.6 | 40.6 | 81.3* |
| Malignant | 33 | 3.1 | 36.3 | 39.4* |
| Total | 65 | 21.5 | 38.5 | 60.0 |

*$p < 0.005$, C.E. = complete effect, P.E. = partial effect

TABLE IV Effectivity rate of Nd-YAG laser treatment according to laser type

| Diagnosis | Patient no. | C.E. (%) | P.E. (%) | Overall (%) |
|---|---|---|---|---|
| Non-contact | 48 | 25.0 | 37.5 | 62.5* |
| Contact | 17 | 11.7 | 41.1 | 52.9* |
| Total | 65 | 21.5 | 38.5 | 60.0 |

*$p > 0.4$, C.E. = complete effect, P.E. = partial effect

Due to its low morbidity and mortality rates, the Nd-YAG laser remained a safe and effective treatment modality and is specifically recommended for lesions causing tracheobronchial obstruction.

### Acknowledgments

We thank Tsung-Tsung Tsai, M.D. for the editorial assistance and for the preparation of the manuscript.

Presented in part at the 19th Congress of the Japanese Society for Bronchology, May 22–24, 1996.

### References

[1] Oho, K., and Ameniya, R. (1988). Endoscopic Nd-YAG laser treatment in airway diseases. In: Joffe, S. N., and Oguro, Y. (eds). *Advances in Nd-YAG Laser Surgery* (Springer-Verlag: New York).
[2] Hetzel, M. R. (1987). Therapeutic aspects of bronchoscopy. In: Dubeis, R. M., and Clarke, S. W. (eds). *Fiberoptic bronchoscopy in diagnosis and management* (Gower Medical Publishing: London).
[3] Daikuzono, N., and Joffe, S. N. (1985). Artificial sapphire probe for contact photocoagulation and tissue vaporization with the Nd-YAG, *Med. Instrum.*, **19**, 173–178.
[4] Toty, L., Personne, C., Colchen, A. *et al.* (1981). Bronchoscopic management of tracheal lesions using the Nd-YAG laser, *Thorax*, **36**, 175–178.
[5] Shapshay, S. M., and Simpson, G. J. (1983). Laser in bronchology, *Otolaryngol. Clin North Amer.*, **16**, 879–887.
[6] Dierkesmmen, R. and Huzly, A. (1985). Side effects of endobronchial laser treatment, *Endoscopy*, **17**, 49–53.
[7] Dumon, J. F., Shapshay, S., Bourcerean, J. *et al.* (1984). Principle for safety in application of Nd-YAG laser in bronchoscopy, *Chest*, **86**, 163–168.
[8] Cavaliere, S., Foccoli, P. and Farina, P. L. (1988). Nd-YAG laser bronchoscopy. A five-year experience with 1396 applications in 1000 patients, *Chest*, **94**, 13–21.
[9] Dumon, J. F., Rebound, E., Garbe, L. *et al.* (1982). Treatment of tracheobronchial lesions by laser photoresection, *Chest*, **81**, 178–184.
[10] Personne, C., Colchen, A. and Leroy, M. (1986). Indications and techniques for endoscopic laser resection on bronchology, *J Thorac Cardiovasc Surg*, **91**, 710–715.
[11] Sutedja, G. and Postmus, P. E. (1994). Bronchoscopic treatment of lung tumor, *Lung Cancer*, **11**, 1–17.

